# Robotic-assisted navigated minimally invasive pedicle screw placement in the first 100 cases at a single institution

**DOI:** 10.1007/s11701-019-00959-6

**Published:** 2019-04-23

**Authors:** Kade T. Huntsman, Leigh A. Ahrendtsen, Jessica R. Riggleman, Charles G. Ledonio

**Affiliations:** 1 Salt Lake Orthopaedic Clinic, Suite 500, 1160 East 3900 South, Salt Lake City, UT 84124 USA; 2 Musculoskeletal Education and Research Center (MERC), A Division of Globus Medical, Inc., 2560 General Armistead Avenue, Audubon, PA 19403 USA

**Keywords:** Robotic-navigated, Pedicle screw placement, Minimally invasive, Spine surgery

## Abstract

Proper pedicle screw placement is an integral part of spine fusion requiring expertly trained spine surgeons. Advances in medical imaging guidance have improved accuracy. There is high interest in the emerging field of robot-assisted spine surgery; however, safety and accuracy studies are needed. This study describes the pedicle screw placement of the first 100 cases in which navigated robotic assistance was used in a private practice clinical setting. A single-surgeon, single-site retrospective Institutional Review Board-exempt review of the first 100 navigated robot-assisted spine surgery cases was performed. An orthopaedic surgeon evaluated screw placement using plain film radiographs. In addition, pedicle screw malposition, reposition, and return to operating room (OR) rates were collected. Results demonstrated a high level (99%) of successful surgeon assessed pedicle screw placement in minimally invasive navigated robot-assisted spine surgery, with no malpositions requiring return to the OR.

## Introduction

Pedicle screw constructs are widely used for posterior fixation in spinal surgery because of their biomechanical superiority and significant correction. However, safe pedicle screw placement is paramount to achieving successful spine surgery [1]. Specialty training is required to avoid the catastrophic neurovascular complications of misplaced screws, which occur in about 4.2% of patients [2]. Nevertheless, pedicle screws are widely used in both young and adult patients, with numerous articles documenting a favorable risk-to-benefit ratio for spinal treatment [3].

Various techniques have been used to guide and confirm pedicle screw placement [4]. The use of anatomic landmarks, plain film radiography, fluoroscopic imaging (standard or image guidance), and computed tomography (CT) image guidance are examples of these techniques [5, 6]. The procedure, benefits, and limitations of each method, as well as comparisons between different approaches, have been widely published [7, 8]. Advances in medical imaging have improved the accuracy of pedicle screw placement from fluoroscopic-guided to computer-aided navigation [8]. The most recent advancement is the use of a navigated robotic-assisted spine surgery system designed to increase the accuracy of pedicle screw placement compared to freehand placement. Clinical outcome studies are required to determine pedicle screw placement accuracy when minimally invasive navigated robotic-assisted spine surgery was performed on the first 100 patients at a single institution.

## Methods

An Institutional Review Board-exempt retrospective chart review was conducted from October 2017 to September 2018 on the first 100 navigated robotic-assisted spine surgeries. The demographic, intraoperative, and perioperative data of 100 patients who underwent lumbosacral pedicle screw placement with minimally invasive navigated robotic guidance using preoperative or intraoperative CT were analyzed. Pedicle screw malposition and reposition rates based on the surgeon’s intraoperative radiographic observations were collected. Secondary outcome measures included patient demographics, robot workflow, length of surgery, length of hospital stay, and intraoperative blood loss.

### Navigated robot-assisted pedicle screw positioning system

**Fig. 1:**
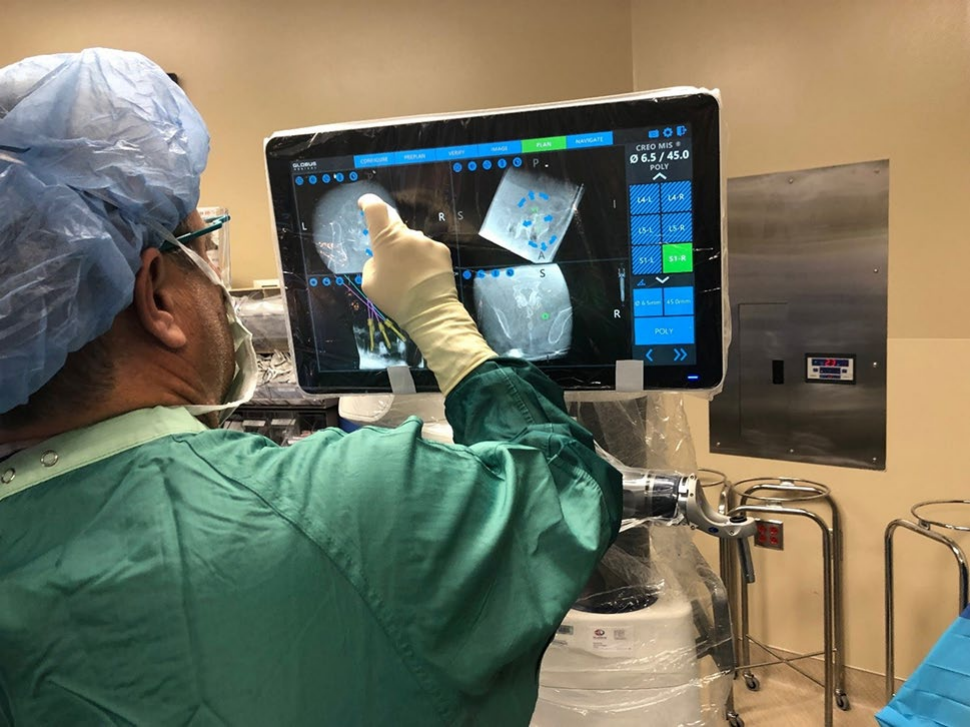
Screw planning with the robotic positioning system

The robotic positioning system (Excelsius GPS^®^; Globus Medical, Inc. Audubon, PA, USA) (Fig. 1) uses either preoperative CT, intraoperative CT, or fluoroscopy, along with a patient reference base and positioning camera to guide pedicle screw placement in real time.

### Preoperative CT workflow

A computed tomography (CT) scan of the spinal levels in the operative field was taken prior to the patient entering the operating room (OR) and screw placement planning was completed. The CT data set was transferred into the robotic positioning system, and then, registration was completed for vertebral levels.

### Intraoperative CT workflow

In intraoperative CT mode, the image coordinate system was obtained from a portable intraoperative CT (e.g., O-arm, Medtronic SNT, Louisville, CO, USA) or standard CT scan was taken at the time of surgery, with the patient already in position on the OR table. Spinal levels were identified and a CT scan was taken. Pedicle screw trajectories were planned and saved.

### Surgical technique

A surgeon-controlled foot pedal activated and positioned the robot arm to the planned pedicle trajectory. Stab incisions were made on the skin using a scalpel. Pedicle screws were inserted under neuromonitoring using navigated instruments guided by the robotic arm. This sequence was repeated until all pedicle screws had been placed. Rods were then placed in a standard fashion. Locking caps were set once the rods were in their proper position. Intraoperative fluoroscopy images were taken to verify the screw and rod position. Pedicle screw placement was qualitatively assessed using postoperative X-rays. Following screw and rod placement, lumbar interbody fusion was performed using 1 of 3 approaches: lateral, anterior, and posterior. The endplates were prepared and the interbody spacer was manually inserted. In the lateral approach, pedicle screws were placed, while patient remained in the lateral decubitus position. In ALIF, patient was repositioned from supine to prone position after interbody placement from the anterior approach. Screws and rods were then placed for posterior supplemental fixation.

### Statistical analysis

Statistical analysis was performed using SPSS Statistics Version 25 software (IBM, Armonk, NY, USA). Data were presented as mean ± standard deviation. The level of statistical significance was set to *p *< 0.05 for all statistical analysis.

## Results

**Fig. 2:**
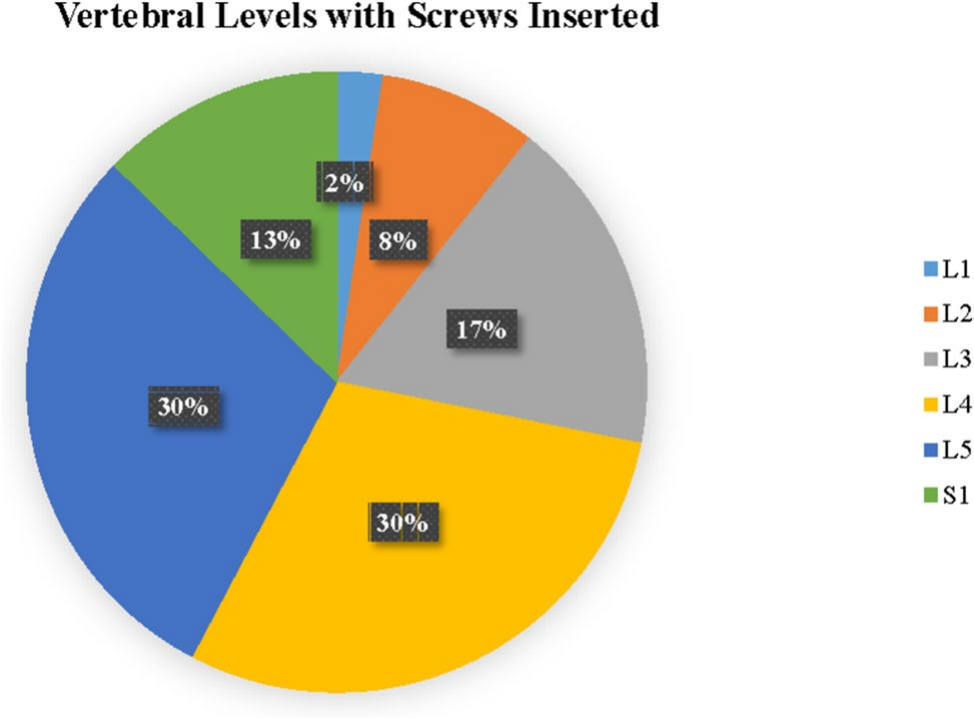
A pie chart depicts the breakdown of vertebral levels among 100 spinal surgery cases. The most common levels with screws inserted are L4 and L5

**Fig. 3:**
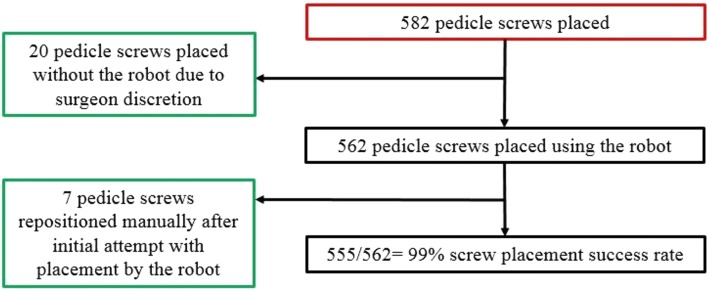
A consortium diagram shows the overall distribution of 100 spinal surgeries using navigated robotic-assisted guidance and pedicle screws. A total of 582 pedicle screws were placed. Five-hundred and sixty-two pedicle screws were placed using the robot. Seven of the 562 pedicle screws were repositioned manually after the initial insertion attempt with the robot. Twenty pedicle screws were placed without the robot due to surgeon discretion

In the first 100 robotic cases, the average age was 63 ± 8 years and 48% (48/100) were female. The average body mass index was 30 kg/m^2^ (range 17–44 kg/m^2^). Twenty-five percent of patients were either current or former smokers. Forty-two percent of patients were retired (Table 1). Of the 100 cases, 55 were lateral lumbar interbody fusion (LLIF), 16 were anterior lumbar interbody fusion (ALIF), and 29 were posterior lumbar interbody fusion (PLIF). The three most common numbers of vertebral levels with pedicle screws inserted were 2-level (36%), 3-level (39%), and 4-level (20%). Intraoperative CT was used in 73 cases, while preoperative CT was used in the remaining 27 cases (Table 2). The most common level with screws inserted was either L4 (30%) or L5 (30%) (Fig. 2). Among the 100 cases, the majority of diagnoses for surgery was degenerative spondylolisthesis with neurogenic claudication (45%) and degenerative spondylolisthesis (17%). A total of 582 pedicle screws were placed. Of the 582 screws, 20 were placed without the robot due to surgeon discretion, leaving 562 pedicle screws inserted by navigated robotic guidance. Of the 562 screws, only 7 had to be repositioned manually due to surgeon discretion to reach a screw placement success rate of 99% (Fig. 3). There were no returns to the OR reported for screw-related complications.

**Table 1:** Baseline characteristics

Parameter	Overall
Number of patients	100
Gender	
Female, *n* (%)	48 (48%)
Male, *n* (%)	52 (52%)
Age, mean (SD, range)	63 (8) (26–82)
BMI, mean (SD, range)	30 (6) (18–44)
Smoker, *n* (%)	
Current	8 (8%)
Former	17 (17%)
Never	75 (75%)
Work status, *n* (%)	
Retired	42 (42%)
Full time	41 (41%)
Part time	7 (7%)
Unemployed	4 (4%)
Disabled	3 (3%)
Unknown	3 (3%)

**Table 2:** Procedure characteristics

Parameter	Overall
Type of surgery, *n* (%)	
LLIF	55 (55%)
PLIF	29 (29%)
ALIF	16 (16%)
Number of levels with screws inserted, *n* (%)	
1	1 (1%)
2	36 (36%)
3	39 (39%)
4	20 (20%)
5	3 (3%)
6	1 (1%)
Workflow, *n* (%)	
Preoperative CT	27 (27%)
Intraoperative CT	73 (73%)

## Discussion

Navigated robotic-assisted spine procedures are in the early development [9]. In this study, a 99% screw placement success rate was reported for pedicle screw placement using minimally invasive navigated robotic-assisted spine surgery. In contrast, the pedicle screw accuracy rate reported by Kosmopoulos and Schizas [10] in a review of 130 studies with 37,337 pedicle screws was 95% with navigation and 90% without navigation [11]. According to Tang et al. [12], pedicle screw placement is variable even with new technologies; however, when compared to freehand screw placement, computer-navigated screws had substantially less risk of cortical damage [13]. Some inaccuracies may be attributed to a learning curve and adapting to a new workflow such as three-dimensional (3D) navigation. The 99% pedicle screw placement success rate using navigated robotic guidance recorded in the current study of the first 100 cases seems to indicate an extremely short learning curve.

Technological advances including navigation have improved the safety and accuracy of pedicle screw fixation. In a meta-analysis by Mason et al. [14], data were gathered from over 30 studies analyzing 9000 pedicle screws and found that the traditional fluoroscopy had an accuracy of 63.1%, two-dimensional navigation had 84.3% accuracy, and 3D navigation was most accurate at 95.5%. Gelalis et al. [15] performed similar analyses and concluded that navigation provides pedicle screw placement with higher accuracy. Jin et al. [16] reported a malposition rate of 9.8% in a series of 1145 screws placed with an intraoperative 2D/3D imaging navigation system.

This initial study of the first 100 cases at a single institution in the clinical use of navigated, robot-assisted spine surgery demonstrated a high pedicle screw placement success rate. There were no postoperative screw malpositions requiring a return to the OR. While this is a single-surgeon, single-site retrospective study, the pedicle screw placement success rate is better than the rates reported in the literature using robot-assisted techniques.

## Conclusion

Navigated robotic guidance provides successful pedicle screw placement at a rate of 99% at this single institution, with a 0% return to OR rate.
